# Energetic Consequences of Human Sociality: Walking Speed Choices among Friendly Dyads

**DOI:** 10.1371/journal.pone.0076576

**Published:** 2013-10-23

**Authors:** Janelle Wagnild, Cara M. Wall-Scheffler

**Affiliations:** 1 Department of Biology, Seattle Pacific University, Seattle, Washington, United States of America; 2 Department of Anthropology, University of Washington, Seattle, Washington, United States of America; University of Illinois at Champaign-Urbana, United States of America

## Abstract

Research has shown that individuals have an optimal walking speed–a speed which minimizes energy expenditure for a given distance. Because the optimal walking speed varies with mass and lower limb length, it also varies with sex, with males in any given population tending to have faster optimal walking speeds. This potentially creates an energetic dilemma for mixed-sex walking groups. Here we examine speed choices made by individuals of varying stature, mass, and sex walking together. Individuals (N = 22) walked around a track alone, with a significant other (with and without holding hands), and with friends of the same and opposite sex while their speeds were recorded every 100 m. Our findings show that males walk at a significantly slower pace to match the females’ paces (p = 0.009), when the female is their romantic partner. The paces of friends of either same or mixed sex walking together did not significantly change (p>0.05). Thus significant pace adjustment appears to be limited to romantic partners. These findings have implications for both mobility and reproductive strategies of groups. Because the male carries the energetic burden by adjusting his pace (slowing down 7%), the female is spared the potentially increased caloric cost required to walk together. In energetically demanding environments, we will expect to find gender segregation in group composition, particularly when travelling longer distances.

## Introduction

In animals, such as humans, that travel substantial distances over ground, we expect selection to have led to an efficient locomotor system (morphology, physiology and behavior), which allows the individual to spend as little energy (and often as little time) as possible on mobility. Those species that adopt effective and efficient mobility strategies not only have time available for other tasks not directly related to mobility (e.g. tool development [1] and socializing [2]–[4]) but also protect the fertility of individuals within the population [5]. There is ample evidence for this energetic trade-off. Daily walking distances have clearly been shown to influence inter-birth-intervals (IBI) and offspring survivorship, with higher distances (and heavier loads) increasing IBIs [6]–[9]. Additionally, high energetic outputs (potentially caused by walking great distances) decrease fertility [10]–[13] particularly if not effectively compensated for by energetic inputs. Therefore, reducing the energetic cost of mobility is highly favorable so that energy is available not only to pay for survival but also to pay for reproduction.

Humans in particular have a number of possible strategies they can employ in order to minimize their cost of mobility. Because humans have a curvilinear relationship between cost of transport (CoT: the metabolic cost to travel a given distance) and speed during both walking and running [14]–[18], there is a speed humans can travel that minimizes their energy expenditure (See Figure S1). Thus, deviation from this ‘optimal speed’ results in higher travel costs. In the interest of minimizing energetic output in order to maintain fertility, it is favorable for individuals to walk at or near their optimum speed and when walking alone, this is the general pattern that humans follow [14], [19]–[22].

An individual’s optimal speed has been shown to correlate strongly with mass and lower limb length, with longer lower limbed and larger individuals having faster optimal speeds [17]. Because of this, people of different masses and/or lower limb lengths will have different optimal speeds. For humans traveling together this can pose significant problems. For instance, human sexual dimorphism in mass and lower limb length [23] leads to male and female differences in optimal speeds, with males having faster speeds than females [17]. If males and females are traveling together, it is impossible for all individuals to be walking at their own optima. In order to walk together, someone must pay the energetic penalty of deviating from his or her optimal speed in order to travel at the same speed as the other individual(s).

It has been suggested that dyad walking speed is correlated to relationship status, such that more intimate relationships yield closer interpersonal distances [24] during walking which causes both individuals to walk more slowly than casual acquaintances [25]. Thus, if male and female couples walk together, they may walk at significantly slower walking speeds than walking alone or with other acquaintances. This could potentially lead to an energetic impact for both sexes.

Since the consequences for such an energetic penalty have reproductive ramifications, understanding how people make decisions when walking together is a key aspect of interpreting human mobility strategies both of living and extinct populations. Here we test the speed choices that individuals make when walking alone versus walking with another individual of the same or different sex. We hypothesize that males will walk faster than females when traveling alone, but that any speed changes when males and females walk together will be influenced by dyad relationship.

## Methods

Eleven males and 11 females (age range 18–29, mean: 22.5±3.8) signed written informed consent forms approved by Seattle Pacific University’s IRB Committee. Seattle Pacific University’s IRB specifically approved the study design presented here. These participants represented couples, such that each male participant (“Male Partner”) was dating or married to a female participant (“Female Partner”) (i.e. 11 couples). At the beginning of the trial, each Partner was asked to walk one lap around a track (400 m) individually at a self-selected pace, while speed was collected using a stopwatch every 100 meters. The average speed for the entire 400 m was then used as each Partner’s baseline for the remainder of the experiment.

Following a period of rest, one Partner (determined by coin toss) was asked to walk continuously around a track. The walking regime consisted of 200 m periods of time walking alone, interspersed with 400 m periods of walking with someone else. In other words, between 400 m of walking with someone else, the Partner walked 200 meters alone for recalibration. In all trials, speed was recorded every 100 meters with a stopwatch. Participants were always asked to walk in the same lane so that they were both walking exactly 400 meters. During the recalibration periods, the speed was within 1% of the initial 400 m lap. All conditions were done in a random order; the orders of variables were generated using http://www.random.org for every Partner and group.

“Walking with someone else” included 400 m with the other Partner and 400 m with the other Partner while holding hands. Holding hands was included as a condition because it is a behavior extensively used by intimate dyads and because it interferes with normal arm swing which has the potential to significantly alter gait in a way specific to romantic partners (or parents). To control for whether relationship status might influence walking speed, friends of the Partners were asked to walk as well. Thus, in addition to walking with the other Partner with and without holding hands, each Partner walked 400 m with a Friend of the same sex and 400 m with a Friend the opposite sex.

In sum, each Partner walked 6 laps per trial (4 variables with 1 lap each, with a solitary half-lap between each variable). After a period of rest, the experiment was repeated with the other Partner. Unfortunately, not all Partners had Friends with whom they could walk; data were collected on only the Partner dyads in those cases. Of the 11 couples who participated, 6 had “Male Friends” of the Male Partners and 8 had “Female Friends” of the Female Partners (age range 18–29, mean: 23.1±4.3). In sum then, the sample consists of 11 Male Partners, 6 Male Friends, 11 Female Partners and 8 Female Friends.

Since the Partners were walking 2.4 km consecutively during each trial and since the range of ambient temperatures was large (see below), it is possible that fatigue and/or weather could potentially affect walking speed throughout the course of each trial. To assess the possible effects of heat and fatigue, the recalibration laps of each Partner were analyzed. Regardless of temperature or distance walked, recalibration speeds were still within 1% of the initial 400-meter lap, thus indicating that neither weather nor fatigue impacted changes in the walking speeds of the Partners within each trial. We suggest that this has to do with the physiological ‘tuning’ related to choosing to walk at one’s optimal walking speed [26].

Basic anthropometrics of all participants were taken, including mass, stature, and lower limb length (greater trochanter to lateral malleolus) (Table 1). In all three measures, males were significantly bigger than females (p<0.001). In these measures, Male Partners and Male Friends were not significantly different (p>0.4), and Female Partners and Female Friends were not significantly different (p>0.3). Lengths of relationships between Partners (mean: 2.2 years) and between Partners and Friends (mean: 3.25 years) were recorded (Table 2). Temperature (range: 1.7–31.7°C, mean: 20.0°C ±11.2°C) and weather conditions (sunny, partly cloudy, cloudy, and rainy) were also noted (Table 3), though did not have a significant impact on any of the results below. The statistics below are independent t-tests when comparing between groups (e.g. males versus females) and paired t-tests when comparing different tasks each group performed (e.g. Male Partners walking alone versus Male Partners walking with Female Partners); all statistics were done in SPSS 18.

**Table 1:** Mean anthropometrics.

Anthropometrics	Male Partners (n = 11)	Mean (std dev)Female Partners (n = 11)	Male Friends (n = 6)	Female Friends (n = 8)
Mass (kg)	81.9 (8.6)	57.2 (6.0)	83.7 (9.1)	57.9 (6.2)
Stature (cm)	184.3 (8.5)	166.9 (7.2)	185.7 (7.2)	164.1 (6.5)
Lower limb length (cm)	79.6 (3.5)	70.1 (1.3)	80.3 (2.6)	69.5 (1.3)

**Table 2:** Mean relationship lengths.

RelationshipLength (Years)	Male Partners	Female Partners
Male Partner	–	2.2 (2.0)
Female Partner	2.2 (2.0)	–
Male Friend	1.0 (1.4)	2.2 (2.9)
Female Friend	2.3 (2.7)	5.5 (7.8)

**Table 3:** Climate and time of day during data collection.

Group	Time of Day	Temperature (°C)	Weather
1	4∶00 p.m.	23.9	Sunny
2	4∶00 p.m.	27.2	Sunny
3	4∶00 p.m.	27.2	Sunny
4	3∶30 p.m.	31.1	Sunny
5	3∶30 p.m.	31.1	Sunny
6	7∶30 p.m.	31.7	Clear Skies (Dusk)
7	2∶00 p.m.	21.7	Partly Cloudy
8	3∶00 p.m.	7.8	Cloudy
9	11∶00 a.m.	1.7	Rainy/snowy
10	2∶00 p.m.	8.3	Partly Cloudy/Windy
11	2∶00 p.m.	8.3	Partly Cloudy/Windy

## Results

### Partners

**Figure 1:**
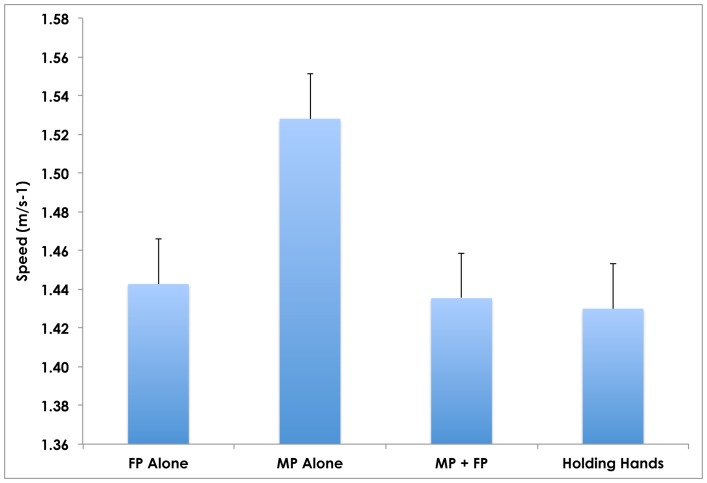
Mean walking speeds of Female Partner (FP) and Male Partner (MP) alone, together, and holding hands. The average walking speed of the MPs significantly slowed to walk with the FP (by 6.3%; p = 0.009) and to hold hands (by 7.0%; p = 0.007), while the FPs’ speeds changed by <1% across all three conditions (alone, with MP, and holding hands). The error bars represent standard error.

Male Partners had faster preferred speeds when walking alone (average: 1.53 ms^−1^) than the Female Partners (average: 1.44 ms^−1^; p = 0.05) (Table 4; Figure 1). When Male and Female Partners walked together, the Male Partners significantly slowed their paces in order to walk with their Female Partners (average: 1.44 ms^−1^; p = 0.009). When asked to hold hands while walking with their Partner, the Male Partners slowed their paces further (average: 1.43 ms^−1^; p = 0.007). The walking speed of the Female Partners only slightly changed when walking with the Male Partners, with or without hand-holding (<1% change compared to Female Partner speed when walking alone). Thus in both cases, the Male Partners nearly matched the preferred speeds of the Female Partners and demonstrated a significant change in their preferred walking speed.

**Table 4:** Mean walking speeds. Mean (standard deviation).

Walking Speeds (ms^−1^)	Male Partners (n = 11)	Female Partners (n = 11)
Alone	1.53 (0.18)	1.44 (0.16)
Male Partner	–	1.44 (0.13)
Male Partner (Holding Hands)	–	1.43 (0.14)
Female Partner	1.44 (0.13)	–
Female Partner (Holding Hands)	1.43 (0.14)	–
Male Friend	1.60 (0.03)	1.48 (0.15)
Female Friend	1.47 (0.15)	1.39 (0.12)

### Opposite-sex Friends

**Figure 2:**
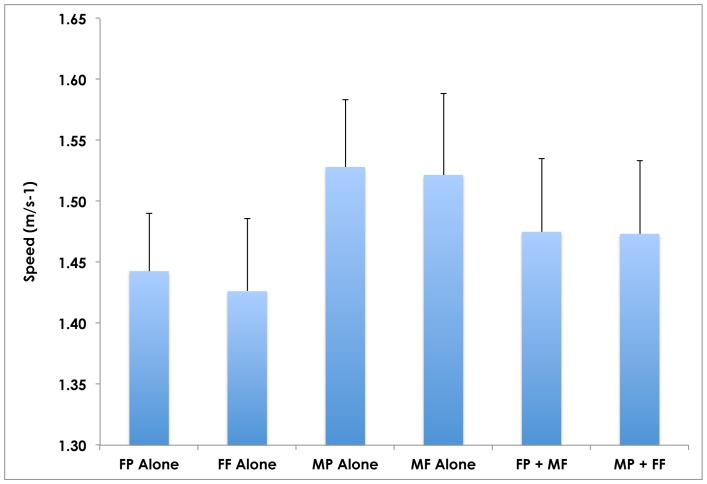
Mean walking speeds of females and males walking alone and in opposite-sex partner-friend pairs. The average walking speed in such dyads demonstrates a compromise of speeds in which the FP sped up by 2.8% (p>0.4) while the MF slowed down by 2.6% (p>0.2) and the FF sped up by 2.9% (p>0.4) while the MP slowed down by 4.0% (p = 0.146). The walking speeds of Partners is also included for comparison (see also Fig. 1). The error bars represent standard error.

When the Female Partners walked with the Male Friends, the Female Partners increased their speeds (from 1.44 ms^−1^ to 1.48 ms^−1^; 2.8%; p = 0.410) while the Male Friends decreased their speeds (from 1.52 ms^−1^ to 1.48 ms^−1^; 2.6%; p = 0.255), thus demonstrating a compromise of speeds (Figure 2). Similarly, when the Male Partners walked with the Female Friends, the Female Friends increased their speeds (from 1.41 ms^−1^ to 1.47 ms^−1^; 4.3%; p = 0.391) while the Male Partners decreased their speeds (from 1.53 ms^−1^ to 1.47 ms^−1^; 4.0%; p = 0.146), again demonstrating a compromise of speeds. In summary, when males and females who were not romantically involved walked together, there was not a significant difference in either’s walking speeds away from solo walking. Males did not significantly slow their speeds to walk with females who were their Friend, though their speed choice did decrease slightly.

### Same-sex Friends

When Male Partners walked with Male Friends, walking speeds were faster than either individual’s preferred walking speed (4%; p = 0.716 for Male Partners and p = 0.595 for Male Friends). When Female Partners walked with Female Friends, walking speeds were slower than either individual’s preferred speed (3%; p = 0.351 for Female Partners and p = 0.571 for Female Friends) (see Figures S2 and S3).

## Discussion

These results are consistent with other data that demonstrate that males walk faster than females both while walking alone [17], and while walking in single sex groups [25], [27]. The results further indicate that any difference in walking speeds between female dyads and male-female dyads is not significant [25]. The data do show however, that there is a decrease in the speed choice between males walking alone and males walking with females; the degree of this speed “accommodation,” however, is linked to the relationship status of the male-female pair, such that males will nearly match the females’ paces only if they are in a romantic relationship. In friendships, the male slows down, but to a lesser (non significant) degree. Furthermore, the differences found between male-male dyads and female-female dyads are also consistent with the hypothesis that social closeness will be mirrored by speed choices [25]. Previous work has noted that women report feeling extremely close to their female friends and here we show that women walk more slowly together even than they do with their Partner. Conversely, men report that they do not feel extremely close or intimate with their male friends and thus here walk more quickly than they do alone [28], [29].

In recent hunter-gatherer populations, males and females often travel similar distances [30] making the energetic consequences of daily mobility an important selection pressure on both sexes. When people of both sexes walk together, either both sexes must pay an energetic penalty by compromising speeds (as seen in the Partner-Friend dyad) or the male must pay an energetic penalty to accommodate the female’s speed (as seen in the Partner-Partner dyad). To alleviate this energetic penalty, many populations travel in single-sex groups in which males travel alone or in pairs and females travel together [31]. By traveling in single-sex groups, there is less variation of body size and optimal walking speeds within the group [17], making this an effective mobility strategy for alleviating the energetic penalty that comes with deviating from one’s own optimum to compromise walking speeds. Additionally, since females seem to have some morphological traits that give them more options in their speed choices [5], [17], [26], even if female ‘closeness’ occurs (and women travel slightly slower than their preferred/optimum), any energetic penalty would be minimal. Alternatively for males, walking away from their energetic optima leads to rapidly increasing energetic costs; if male speeds are going to increase when walking together, the energetic burden could become quite high [17], thus suggesting one possible reason for single-individual male hunting and foraging [5], [17], [26] (other reasons relating to specific hunting and foraging strategies may likely also be of issue).

If males and females are not traveling separately, the males are much more likely to bear the energetic burden in order to walk with the females, particularly if they are partners. From an energetics perspective, this is the expected outcome since the female reproductive system is sensitive to even the slightest energetic perturbations [11], [12], [32]. If a female is in negative energy balance, ovarian function may be stifled, thus eliminating the possibility of conceiving until energy balance is restored by either expending less energy or consuming more energy [11]. Walking great distances is energetically demanding, making it crucial for females to be walking at or near their optima in order to minimize the energetic cost of walking as much as possible so that energy can be allocated to reproduction. The male reproductive system is much more resilient to energetic expenditures so that even high energetic outputs have no clear impact on sperm production [11]. In order to protect the fertility of females, males may bear the energetic penalty to walk at the females’ paces if they are walking together, and are more likely to bear this burden with a romantic partner than with another female.

Work amongst the Hadza demonstrates that males provision for their females following parturition and early lactation periods when females are less productive than at other times [33], thus suggesting an investment in a mate even when she is not fertile and/or less sexually desirable. This exemplifies the willingness and capability of males to expend energy in order to obtain and allocate energetic resources for the female in whom he is reproductively invested. Within a mobility context, the male pays the energetic cost of deviating from his optimal speed in order to walk at the female’s optimum, allowing her to conserve energetic resources that can be allocated to reproduction. It would be useful in the future to assess how male speed is influence while walking with female partners experiencing reproductive loads. Since pregnancy and lactation are very energetically expensive [26], [34]–[39], mobility costs must be minimized in order to conserve maternal resources and it is even more unlikely that males would not slow down to walk with females. However, females walk significantly slower (partly due to a slower optimal walking speed) when loaded [26]; for males to slow down to the speed women walk while loaded, they potentially increase their cost up to 10% of their daily energy expenditure [17] creating a more significant burden on their resources. Further testing could assess whether this more substantial cost for males encourages a lesser accommodation.

Implications from this study extend beyond extant human populations. Recent fossil discoveries have shown multiperson walking groups [40]–[43] comprised of individuals of varying sizes who were likely traveling at the same speed. Furthermore, different footprint groups seem to show different patterns, with one group showing a few ‘large’ individuals [43] and another showing primarily women with juveniles and children [40]–[42]. These group compositions seem very reasonable given the findings here (i.e. each group traveling within morphological and speed constraints).

## Supporting Information

Figure S1 The cost of transport curve. The data are from Wall-Scheffler & Myers 2013.
(TIF)

Figure S2 Male Partner (MP) and Male Friend (MF) speeds walking alone and together.
(TIF)

Figure S3 Female Partner (FP) and Female Friend (FF) walking speeds alone and together.
(TIF)
