# Supplementary figures and images for: Energetic Consequences of Human Sociality: Walking Speed Choices among Friendly Dyads
Source: PLoS One. 2013 Oct 23;8(10):e76576. doi: 10.1371/journal.pone.0076576 (PMC3806777; doi:10.1371/journal.pone.0076576)

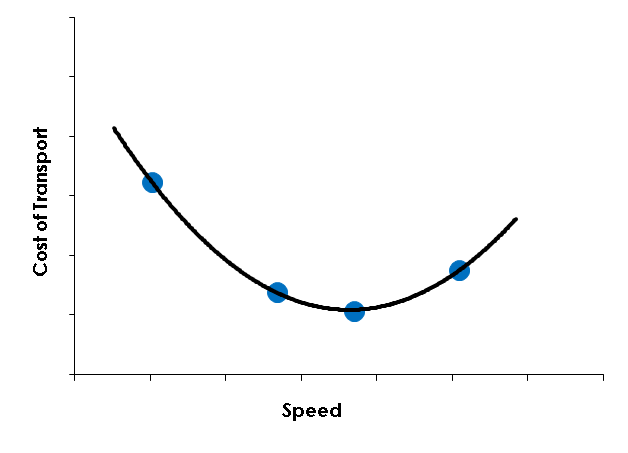

Supplement: Figure S1 — The cost of transport curve. The data are from Wall-Scheffler & Myers 2013. (TIF) [file pone.0076576.s001.tif]

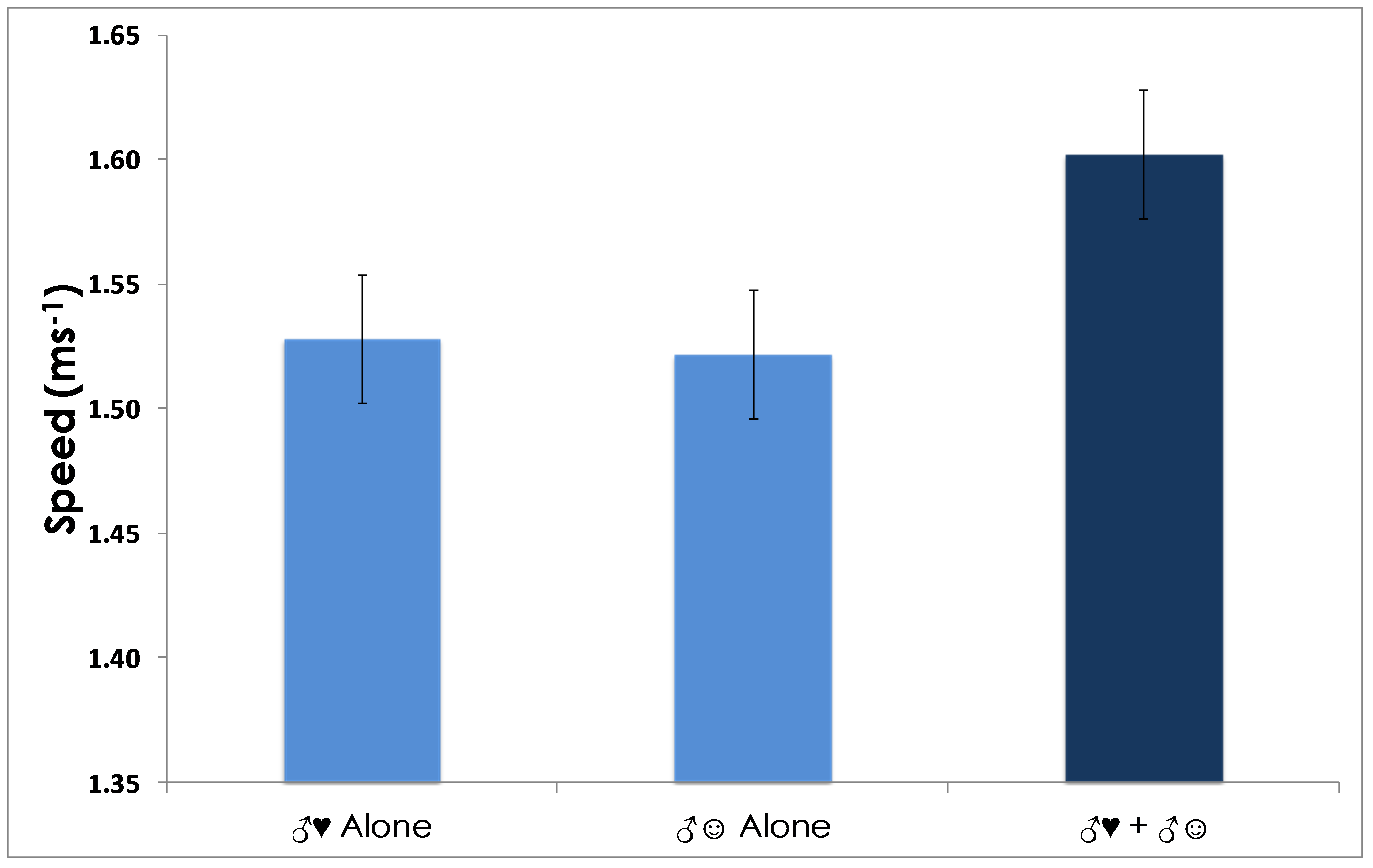

Supplement: Figure S2 — Male Partner (MP) and Male Friend (MF) speeds walking alone and together. (TIF) [file pone.0076576.s002.tif]

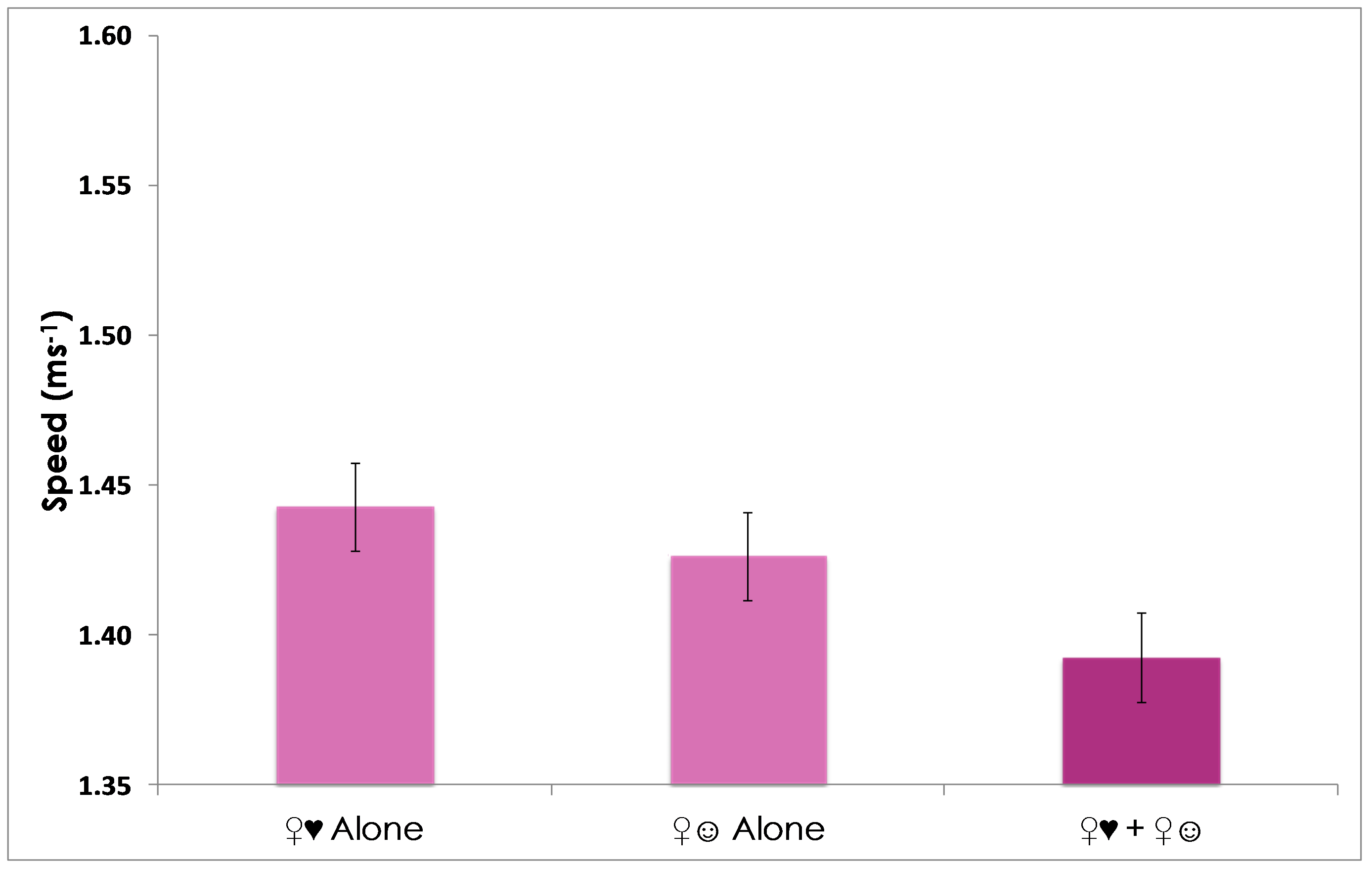

Supplement: Figure S3 — Female Partner (FP) and Female Friend (FF) walking speeds alone and together. (TIF) [file pone.0076576.s003.tif]
